# Glandular Enlargement and Other Diseases of the Lymphatic System

**Published:** 1911-09

**Authors:** 


**Glandular Enlargement and other Diseases of the Lymphatic System.** By Arthur Edmunds, M.B., M.S., B.Sc. Lond., F.R.C.S. Eng. Pp. vii, 230. London: The Oxford Medical Publications. 1908.

Mr. Edmunds in this work has kept within the limits of practical surgery, and has not favoured the reader with much that is new with reference either to the development or to the anatomical distribution of the lymphatic system. He lays stress upon the rôle played by this system in dealing with diseases and in the general economy of the organism.

The subject is arranged under pathological headings, the early chapters dealing with acute infections and inflammations of the vessels and glands, and the subsequent sections taking specific and parasitic infections, and finally simple and malignant enlargements of the lymphatic glands.

The pathology of each class of cases is dealt with very clearly, and in such a way as to render the work a valuable help in the task of diagnosing between the many various forms of lymphatic gland enlargement.

A marked feature of the work is the decided way in which it defines the operable from the inoperable case; and another welcome feature is the way in which the difficulties and dangers of extensive operations on glands in the neck are set forth with suggestions as to how best they may be avoided.

The method of treating tuberculous glands by means of vaccines is not supported by any strong or convincing evidence.

In style the book is readable, and in material it will be found to contain much useful information and many very practical suggestions.

